# Supplementary material for: A novel DNA repair-independent role for Gen nuclease in promoting unscheduled polyploidy cell proliferation
Source: PLoS Genet. 2025 Sep 8;21(9):e1011605. doi: 10.1371/journal.pgen.1011605 (PMC12440172; doi:10.1371/journal.pgen.1011605)
Supplement: Attachment — Submitted filename: Response to Reviewers Budzyk et al 2025.pdf [file pgen.1011605.s008.pdf]

We thank all the reviewers for their enthusiastic reviews. We have addressed all the comments. The answers to review's comments are in blue. The changes in the manuscript are highlighted in yellow- modified text.

Reviewer #1: The study "A novel DNA repair-independent role for Gen nuclease in promoting unscheduled polyploidy cell proliferation" by Budzyk et. al. explores the use of Gen (Gen1/Yen1) in cell proliferation and DNA replication in polyploid cells. They show that Gen is not needed in diploid cells for preventing DNA damage and genome instability, even upon aphidicolin induced replication stress. However, Gen is essential to maintain polyploid cells. Deletion of Gen leads to smaller and slower dividing polyploid cells. Interestingly, overexpression of Gen in diploids and in polyploids leads to ectopic DNA damage. Finally, overexpression of Gen is similar to overexpressed nuclease dead Gen in polyploid cells, indicating that Gen has a nuclease-indepdendent role in maintaining polyploidy.
Overall, it's a well written manuscript. I have a few concerns regarding the manuscript that I outline below.

1. Fig 1G and 3E have multiple statistical tests within the same figure. The authors claim they used Mann-Whitney test.

a. They should mention if it is two-tailed or one-tailed.

We apologize for the mis-understanding here. We used Two-tailed Mann-Whitney tests. In graphs that contain multiple data points (more than two), we used a non-parametric Kruskal-Wallis test. We have added all this information to the figure legends. In doing the analysis for all data, we first submitted the data to normality and log normality tests for the choice of appropriate statistical test.

b. Multiple hypothesis testing is missing, q-values should be calculated and reported.

We have reported p-values, according to what is normally required for publication and these are the ones included in the Figures mentioned above. We have also determined the q-values after two-stage linear step-up procedure of Benjamini, Krieger and Yekutieli. For all the data included in this paper, significance was not changed. We would prefer to just include the p-values, as this is what is normally required and presented in journals.

2. Fig 4B – while it looks obvious, a Chi square goodness of fit test or a similar statistical test should be done to show that the differences in Ctrl, GenOE and Genmut are statistically different.

We have included this information in Fig4B- now 5B. As the reviewer suspected the Ficher's test shows that the differences between Ctrl and Gen^{mut} are highly significative, while the difference between Ctrl and GenOE are significative, but less.

3. In the introduction, it is not clear what the link is between whole genome duplication and HR. The authors should consider re-writing the section just to better explain why HR is needed in polyploid cells.

We have changed the last paragraph of the introduction section to facilitate comprehension. We have also changed the structure of the introduction. All changes are highlighted in yellow.

4. Results – "Using a NB specific GAL4 driver (Worniu-GAL4), we depleted by RNA interference (RNAi) Rad51, BLM, Mus81, SLX1 and Gen in polyploid brains. Rad51 is a major player in HR [9], while the remaining proteins function in Holiday Junction (HJ) processing [12,30]."
BLM also functions in resection and initiation of HR.

The reviewer is absolutely right and we have modified the text to reference to these BLM functions. It is highlighted in yellow.

5. Ctrl WT – this is jargon – please define usage.

We apologize for this. WT stands for Wild type and Ctrl for control. We have modified the text accordingly. . It is highlighted in yellow.

6. The authors show that GenND leads to increased hH2Av in polyploid cells. What about diploids? This is a major control, that should be done.

As suggested by the reviewer, we now included data related with GenND in diploid brains. We characterize both the mitotic index and the levels of DNA damage in brains expressing UAS-GenND specifically in NBs. We did not find any effect of this expression in diploid conditions in terms of DNA damage. But the mitotic index is increased when compared to controls. These results show that the defects in mitosis and ectopic DNA damage typical of UAS-Gen in diploid conditions are dependent on nuclease activity. We have described these – highlighted in yellow and included quantifications in Supplementary Figure 4D-E.

Reviewer #2: In this study the Authors identified a novel, DNA repair-independent role of Gen nuclease in promoting unscheduled polyploidy cell proliferation. Traditionally, Gen is known for its role in DNA damage repair, specifically in Holliday junction resolution during homologous recombination. However, this study demonstrates that

Gen has an unexpected function in regulating DNA replication rates and cell cycle progression in polyploid cells. This is a strong Article that can be published.

Abstract: 1. Please, emphasize the unexpected, DNA repair-independent role of Gen nuclease earlier to immediately capture the reader's attention. Please, strengthen the conclusion.

We understand this reviewer's comment. However, we would prefer not to change the abstract structure as for non-experts it may be difficult to understand if we describe right away Gen's novel role.

Introduction: 1.The introduction should smoothly transition from genome integrity and DNA damage response (DDR) to unscheduled whole genome duplication (WGD) and its implications. Currently, the link between WGD, replication stress, and the necessity for DNA damage repair is implied but not clearly stated. Make the connection more explicit. The final paragraph could more clearly emphasize the knowledge gap you are addressing and how your study contributes.

We have followed this reviewer's suggestion and altered the introduction chapter. The new text is highlighted in yellow.

2. Please, explain benefits of your model using Drosophila neural stem cells- also known as neuroblasts (NBs) that are highly polyploid. It is also would be good to underline the important role of polyploidy in neurodegenerative disorders.

We have included more text to explain better the use of NBs. And we have included the reference of polyploidy in the drosophila ageing brain in the introduction. This is really important.

Discussion: 1. Please, clarify Gen's Role in Diploid Cells; 2. Explain how loss-of-function delays mitosis, while overexpression accelerates it. 3. Highlight mitotic defects (e.g., chromosome bridges, lagging chromosomes). 4. Highlight that Gen is essential for polyploid cell proliferation, with loss-of-function reducing cell size and overexpression increasing proliferation despite DNA damage. 5. Discuss how cell cycle asynchrony and DNA replication rates are affected. 6. Please, discuss therapeutic and Biomedical Implications Emphasize that Gen inhibition selectively affects polyploid cells, making it a potential therapeutic target for polyploid tumors. 7. Propose future directions, including mammalian validation studies and drug development targeting Gen activity.

As requested by this reviewer, we have extended the discussion. The new text is highlighted in yellow. We would prefer, however not to discuss future directions with details and in particular work in human cells, as this is currently one of the subjects being developed in the lab. This represents the work of a PhD student and so discussing potential results concerning human cells, would represent a risk to his PhD.

Reviewer#3

Summary
The integrity of the genome depends on proper DNA replication and chromosome segregation. Unscheduled DNA replication can lead to replication stress and DNA damage, that may even drive tumor evolution via genome duplication. Homologous recombination is one repair pathway that repairs double strand breaks during replication stress, involving the Gen nuclease. The authors use a Drosophila neural stem cell model to investigate the role of Gen, specifically in cycling polyploid/multinucleate cells . Loss of Gen impairs cell cycle progression in polyploid cells, while its over-expression accelerates cell cycles despite increasing DNA damage levels. The authors find that Gen's role in promoting polyploid cell proliferation appears to be independent of its DNA repair and Holliday Junction processing activities. The authors suggests that Gen may have an additional function related to DNA replication during S-phase.

Evaluation

This paper provides compelling insights into the role of Gen nuclease, its little understood role in genome integrity and the biology of polyploid cells. The experimental design and data analysis are robust, the findings are of high quality and I strongly support its publication in PLOS Genetics.

I have minor comments related to clarity of the paper:

Is there a way to sharpen the language regarding use of the term 'polyploid'?
The term "polyploid" is broad and includes classically endocycling cells, such as those in the salivary glands. However, the manuscript deals with multinucleate cells (see comment 2). I do understand that the pitch of the story is more general, but I also think that polyploid or multinucleate is an important distinction, that if made, may help the paper in the long run.

We fully understand this reviewer's point and we have therefore defined the concept of polyploidy better and the fact that we are describing polyploid NBs that were generated by cytokinesis failure. We would like to avoid mentioning binucleation or multinucleation, however. The reason behind this choice relates with the fact that cytokinesis failure initially results in binucleated cells, but after a few cell cycles, the massive accumulation in DNA content results frequently in chromosome collapse at the end of mitosis, making it difficult to assess the number of nuclei. This is certainly due to defects in DNA replication and nuclear asynchrony as published before (Nano et al, 2019 and Gemble et al, 2022). We have explained this point better in the text, both in the introduction and in the results, which are highlighted in yellow.

Comment 1

'…Here we investigated the function of Gen in an *in vivo* model of unscheduled WGD/polyploidy that generates neural stem cells- also known as neuroblasts (NBs) that are highly polyploid.'..

> Please rewrite this sentence. I had to read it multiple times to understand that you meant to say that your model generates polyploidy, not that neural stem cells are (normally) polyploid.

As suggested by the reviewer we have simplified this sentence in the last paragraph of the introduction. The whole paragraph is highlighted in yellow to include changes requested by another reviewer. This particular sentence is underlined to facilitate identification.

Comment 2

'.. Wild type neural stem cells (also called neuroblasts- NBs) are diploid (Figure 1A), but repeated cytokinesis failure in the hypomorphic mutant *spaghetti squash*- *sqh1*- referred here as *sqhmut*, generates NBs that accumulate polyploid large nuclei (Figure 1B-C), containing multiple copies of all chromosomes[27,28]…'

> But Sqh this polyploidy is multinucleated because of cytokinesis failure. Should the term used not be multinucleate throughout the story to distinguish this type of polyploidy from polyploid by endoreplication.

> *This comment relates with the previous comment made by this reviewer. Polyploidy is defined by the increase in the number of all chromosomes. It can be induced through endoreplication, mitotic bypass, cytokinesis failure or cell fusion. For example, in mammalian hepatocytes, polyploidy is induced through cytokinesis failure. And these cells are described as polyploid. The same for Drosophila sub-peripheral glia. In sqh^{mut} NBs, even if they fail cytokinesis and become binucleated cells, some of them later on will no longer perform anaphase B- we think due to the high number of chromosomes- and form multilobated nuclei. Also, we and others have referred to these as polyploid neuroblasts (Nano et al, 2019, Goupil et al, 2020), so we prefer to keep this nomenclature. We have explained this better in the results (highlighted in yellow) and included more references.*

Comment 3

'…Characterization of polyploid, Rad51RNAi brains, revealed an increase in their mitotic index, suggesting that Polyploid, Rad51RNAi NBs arrest/or are severed delayed in mitosis…'

> I understand this effect but readers not from the cell cycle field may not: Why does a mitotic index increase suggest the cells arrest? Please approach this with an explanation that is already foreshadowing the conclusions for (Figure 2 I-J) – pH3 high can be both slow and fast cell cycles. Maybe you need to rebrand 'mitotic index' because it is so synonoumously used (maybe carelessly) with 'proliferation rate' in the literature?

> *Yes, this is certainly a big problem and we agree with the reviewer that is often mentioned as an indicator of proliferation. We have included a definition of mitotic index and justified our explanation.*

Comment 4

by products

> should probably be spelled byproducts, right?

Yes, absolutely. We have corrected this.

Comment 5

Why is experiments S4 in supplements? I understand you want to maintain the focus of the story on the function of Gen in polyploidy but there is so little known about Gen that I think at least experiment S4I, J could make it into the main figures…

As suggested by the reviewer, we have moved this experiment to the main Figure. It is now Figure 3.
