# Supplementary material for: A novel DNA repair-independent role for Gen nuclease in promoting unscheduled polyploidy cell proliferation
Source: PLoS Genet. 2025 Sep 8;21(9):e1011605. doi: 10.1371/journal.pgen.1011605 (PMC12440172; doi:10.1371/journal.pgen.1011605)
Supplement: Attachment — Submitted filename: Budzky & Basto 2025.docx [file pgen.1011605.s006.docx]

**Summary**

The integrity of the genome depends on proper DNA replication and chromosome segregation. Unscheduled DNA replication can lead to replication stress and DNA damage, that may even drive tumor evolution via genome duplication. Homologous recombination is one repair pathway that repairs double strand breaks during replication stress, involving the Gen nuclease. The authors use a Drosophila neural stem cell model to investigate the role of Gen, specifically in cycling polyploid/multinucleate cells . Loss of Gen impairs cell cycle progression in polyploid cells, while its over-expression accelerates cell cycles despite increasing DNA damage levels. The authors find that Gen’s role in promoting polyploid cell proliferation appears to be independent of its DNA repair and Holliday Junction processing activities. The authors suggests that Gen may have an additional function related to DNA replication during S-phase.

**Evaluation**

This paper provides compelling insights into the role of Gen nuclease, its little understood role in genome integrity and the biology of polyploid cells. The experimental design and data analysis are robust, the findings are of high quality and I strongly support its publication in PLOS Genetics.

I have minor comments related to clarity of the paper:

Is there a way to sharpen the language regarding use of the term ‘polyploid’?

The term "polyploid" is broad and includes classically endocycling cells, such as those in the salivary glands. However, the manuscript deals with multinucleate cells (see comment 2). I do understand that the pitch of the story is more general, but I also think that polyploid or multinucleate is an important distinction, that if made, may help the paper in the long run.

**Comment 1**

‘…Here we investigated the function of Gen in an *in vivo* model of unscheduled WGD/polyploidy that generates neural stem cells- also known as neuroblasts (NBs) that are highly polyploid.’..

Please rewrite this sentence. I had to read it multiple times to understand that you meant to say that your model generates polyploidy, not that neural stem cells are (normally) polyploid.

**Comment 2**

‘.. Wild type neural stem cells (also called neuroblasts- NBs) are diploid (Figure 1A), but repeated cytokinesis failure in the hypomorphic mutant *spaghetti squash- sqh1*- referred here as *sqhmut*, generates NBs that accumulate polyploid large nuclei (Figure 1B-C), containing multiple copies of all chromosomes[27,28]…’

But Sqh this polyploidy is multinucleated because of cytokinesis failure. Should the term used not be multinucleate throughpout the story to distinguish this type of polyploidy from polyploid by endoreplication.

**Comment 3**

‘…Characterization of polyploid, Rad51RNAi brains, revealed an increase intheir mitotic index, suggesting that Polyploid, Rad51RNAi NBs arrest/or are severed delayed in mitosis…’

I understand this effect but readers not from the cell cycle field may not: Why does a mitotic index increase suggest the cells arrest? Please approach this with an explanation that is already foreshadowing the conclusions for (Figure 2 I-J) – pH3 high can be both slow and fast cell cycles. Maybe you need to rebrand ‘mitotic index’ because it is so synonoumously used (maybe carelessly) with ‘proliferation rate’ in the literature?

**Comment 4**

by products

should probably be spelled byproducts, right?

**Comment 5**

Why is experiments S4 in supplements? I understand you want to maintain the focus of the story on the function of Gen in polyploidy but there is so little known about Gen that I think at least experiment S4I, J could make it into the main figures…
